# Symptomatic Intracranial Hemorrhage After Endovascular Stroke Treatment: External Validation of Prediction Models

**DOI:** 10.1161/STROKEAHA.122.040065

**Published:** 2023-01-24

**Authors:** Nadinda A.M. van der Ende, Femke C.C. Kremers, Wouter van der Steen, Esmee Venema, Manon Kappelhof, Charles B.L.M. Majoie, Alida A. Postma, Jelis Boiten, Ido R. van den Wijngaard, Aad van der Lugt, Diederik W.J. Dippel, Bob Roozenbeek

**Affiliations:** 1 Departments of Neurology (N.A.M.v.d.E, F.C.C.K., W.v.d.S, E.V., D.W.J.D., B.R.), Erasmus MC University Medical Center, the Netherlands.; 2 Radiology and Nuclear Medicine (N.A.M.v.d.Ee, W.v.d.S., B.R.), Erasmus MC University Medical Center, the Netherlands.; 3 Emergency Medicine (E.V.), Erasmus MC University Medical Center, the Netherlands.; 4 Department of Radiology and Nuclear Medicine (M.K.), Amsterdam UMC, University of Amsterdam, the Netherlands.; 5 Department of Radiology and Nuclear Medicine, School for Mental Health and Sciences, Maastricht University Medical Center, the Netherlands (A.A.P.).; 6 Departments of Neurology (J.B., I.R.v.d.W.), Haaglanden Medical Center, the Netherlands.; 7 Radiology and Nuclear Medicine (I.R.v.d.W.), Haaglanden Medical Center, the Netherlands.

**Keywords:** endovascular treatment, ischemic stroke, symptomatic intracranial hemorrhage

## Abstract

Symptomatic intracranial hemorrhage (sICH) is a severe complication of reperfusion therapy for ischemic stroke. Multiple models have been developed to predict sICH or intracranial hemorrhage (ICH) after reperfusion therapy. We provide an overview of published models and validate their ability to predict sICH in patients treated with endovascular treatment in daily clinical practice.

**Methods:**

We conducted a systematic search to identify models either developed or validated to predict sICH or ICH after reperfusion therapy (intravenous thrombolysis and/or endovascular treatment) for ischemic stroke. Models were externally validated in the MR CLEAN Registry (n=3180; Multicenter Randomized Clinical Trial of Endovascular Treatment for Acute Ischemic Stroke in the Netherlands). The primary outcome was sICH according to the Heidelberg Bleeding Classification. Model performance was evaluated with discrimination (c-statistic, ideally 1; a c-statistic below 0.7 is considered poor in discrimination) and calibration (slope, ideally 1, and intercept, ideally 0).

**Results:**

We included 39 studies describing 40 models. The most frequently used predictors were baseline National Institutes of Health Stroke Scale (NIHSS; n=35), age (n=22), and glucose level (n=22). In the MR CLEAN Registry, sICH occurred in 188/3180 (5.9%) patients. Discrimination ranged from 0.51 (SPAN-100 [Stroke Prognostication Using Age and National Institutes of Health Stroke Scale]) to 0.61 (SITS-SICH [Safe Implementation of Treatments in Stroke Symptomatic Intracerebral Hemorrhage] and STARTING-SICH [STARTING Symptomatic Intracerebral Hemorrhage]). Best calibrated models were IST-3 (intercept, −0.15 [95% CI, −0.01 to −0.31]; slope, 0.80 [95% CI, 0.50−1.09]), SITS−SICH (intercept, 0.15 [95% CI, −0.01 to 0.30]; slope, 0.62 [95% CI, 0.38−0.87]), and STARTING−SICH (intercept, −0.03 [95% CI, −0.19 to 0.12]; slope, 0.56 [95% CI, 0.35−0.76]).

**Conclusions:**

The investigated models to predict sICH or ICH discriminate poorly between patients with a low and high risk of sICH after endovascular treatment in daily clinical practice and are, therefore, not clinically useful for this patient population.

Reperfusion therapy (ie, intravenous thrombolytics (IVT), endovascular thrombectomy (EVT), or a combination of both) is an effective treatment for ischemic stroke at group level, despite the increased average risk of symptomatic intracranial hemorrhage (sICH).^1,2^ On an individual level, the occurrence of sICH after reperfusion therapy increases the likelihood of poor functional outcome and death.^3^ Reliable identification of individual patients with high risk of sICH could be useful to clinicians when therapeutic decisions are made, to inform patients and relatives on prognosis, and to personalize monitoring protocols.^4^

Several prediction models that aim to identify patients with a high risk of sICH after reperfusion therapy have been published.^5–11^ Before a prediction model can be implemented in clinical practice, the model should be evaluated thoroughly. External validation is essential in this evaluation because it assesses the generalizability of the model.^12^ Only a few models to predict sICH have been developed or externally validated in patients receiving EVT for ischemic stroke.^9–11,13^

We aimed to provide an overview of currently published models to predict sICH or ICH after reperfusion therapy and to externally validate their ability to predict sICH in patients treated with EVT in daily clinical practice.

## Methods

### Search Strategy and Eligibility of Prediction Models

A search strategy was developed in collaboration with a biomedical information specialist to systematically search PubMed, Embase, Medline, Web of Science, and Cochrane to identify studies reporting on the development or validation of models based on clinical, radiological and treatment-related variables to predict sICH or any ICH after reperfusion therapy for ischemic stroke. We included studies that included at least 2 variables in the model and were published in peer-reviewed journals. The search was restricted to studies published in English and conference abstracts were excluded. The search was conducted in August, 2021. The complete search strategy is listed in Table S1. Two reviewers (NvdE and FK) independently screened all titles and abstracts of the retrieved references. Subsequently, full-text copies of articles that potentially met the criteria were independently reviewed for final inclusion in this study. Consensus was reached with a third reviewer (DD) when needed.

### External Validation Cohort

We used data from the MR CLEAN Registry (Multicenter Randomized Clinical Trial of Endovascular Treatment for Acute Ischemic Stroke in the Netherlands). The MR CLEAN Registry is a prospective, observational study of all patients who underwent EVT for ischemic stroke in the Netherlands. Details on the MR CLEAN Registry were published previously.^2^ For the present study, we selected patients who were registered between March 16, 2014 and November 1, 2017 and adhered to the following inclusion criteria: age≥18 years; treatment in a center that participated in the MR CLEAN trial; presence of a proximal intracranial occlusion in the anterior circulation confirmed on noninvasive vascular imaging (intracranial carotid artery [internal carotid artery (terminus)], middle cerebral artery [M1/M2], anterior cerebral artery [A1/A2]); and groin puncture within 6.5 hours after symptom onset. The central medical ethics committee of the Erasmus MC University Medical Center Rotterdam, the Netherlands, evaluated the study protocol and granted permission to carry out the study as a registry (MEC-2014-235). In compliance with the General Data Protection Regulation, source data are not available for other researchers. Information about analytic methods, study materials, and scripts of the statistical analyses are available from the corresponding author on reasonable request. The STROBE statement checklist of the study can be found in Table S2.

### Predicted Outcome

We externally validated the models for their performance to predict sICH within 90 days after intervention. An ICH was deemed symptomatic if a patient died or deteriorated neurologically and evidence of related ICH on follow-up imaging (non-contrast computed tomography or magnetic resonance imaging).^14^ To minimize biased reporting, the imaging core laboratory analyzed the follow-up images of patients with sICH and the complication committee made the final decision for reporting a sICH.

### Statistical Analysis

Models including predictors that were not included in the MR CLEAN Registry database were reconstructed from available variables if possible. If this was not possible, these models were included in the overview, but excluded for external validation.

Model performance was evaluated with discrimination and calibration. Discrimination was quantified with the concordance (c) statistic, which is identical to the area under the receiver operating curve for binary outcomes. The c statistic varies between 0.5 for a non-informative model and 1 for a perfectly discriminating model.^15^ A c statistic below 0.7 is considered a poor discriminative ability, a c statistic between 0.7 and below 0.8 is considered acceptable, a c statistic between 0.8 and below 0.9 is considered excellent, and a c statistic of 0.9 and above is considered outstanding.^16^ Calibration refers to the level of agreement between predicted risks and observed outcome expressed as a calibration intercept and slope. The intercept indicates whether predictions are systematically too high or too low, and should ideally be zero. The calibration slope describes the effect of the predictors in the validation sample and is ideally equal to 1.^12^ For models developed to predict outcomes other than sICH or ICH, but that have been evaluated for ICH prediction, calibration was only assessed if the regression model was adapted for ICH prediction.

For models that were presented as a risk score, we used the predicted probabilities of each model as published in the original development article. If a study expressed a probability of 0 (0%) for the outcome of interest, this value was adapted to 0.01 (1%), because these probabilities were otherwise excluded by the *val.prob.ci.2* function in R.^17^ If the predicted probabilities were not published, we contacted the corresponding author to provide the predicted probability of the model. For regression models, authors were contacted to provide the regression formula if this was not reported.

Missing values were imputed with multiple imputation (n=5) using the function *aregImpute*. Confidence intervals of the model performance measures were composed with bootstrapping (200 samples in 5 imputed datasets).

All statistical analyses were performed with R statistical software (version 4.0.5).

## Results

**Figure 1.**
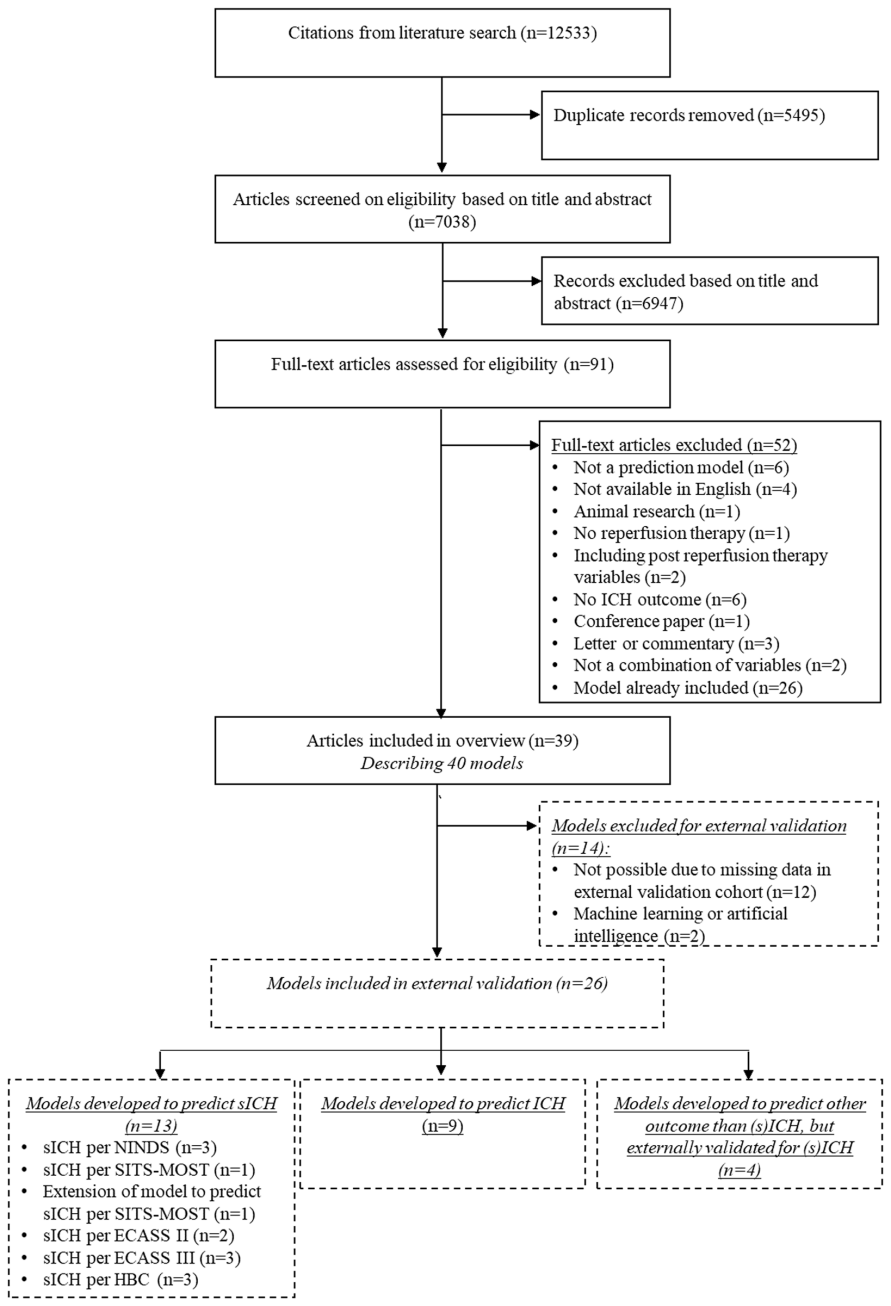
**Flow chart of the systematic literature search.** ECASS indicates European Cooperative Acute Stroke Study; HBC, Heidelberg Bleeding Classification; ICH, intracranial hemorrhage; NINDS, National Institute of Neurological Disorders and Stroke; SICH, symptomatic intracranial hemorrhage; and SITS-MOST, Safe Implementation of Thrombolysis in Stroke-Monitoring Study.

The literature search identified 7038 unique studies. A total of 6947 studies were excluded based on title and abstract. We assessed the full texts of 91 studies and included 39 studies, which described 40 models (Figure 1). Model development characteristics of the included models are shown in Table S3. The number of predictors varied between 2 and 14 predictors. The most frequently used predictors were baseline National Institutes of Health Stroke Scale (NIHSS; n=35), age (n=22), and baseline blood glucose (n=22; Table 1).

**Table 1.**
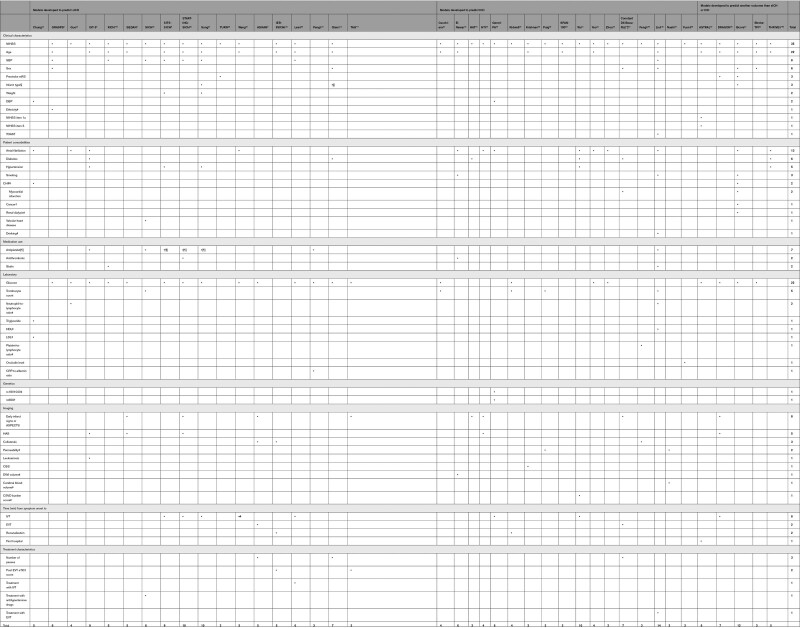
Overview of Predictors Included in the Models

We excluded 14 models for external validation (Figure 1). For 8 included models, 1 predictor was adapted to be able to externally validate the model in the MR CLEAN Registry (Table S4). Of the 26/40 models available for external validation, 7/26 were developed in patients treated with EVT. Calibration of 6 models (GRASPS [GWTG-Stroke sICH Risk], Sung, Kidwell, SPAN-100 [Stroke Prognostication Using Age and National Institutes of Health Stroke Scale], ASTRAL [Acute Stroke Registry and Analysis of Lausanne], DRAGON [dense cerebral artery sign/early infarct signs on admission CT scan, prestroke modified Rankin Scale, age, glucose level at baseline, onset-to-treatment time, and baseline National Institutes of Health Stroke Scale score]) could not be assessed due to missing data in articles.

The external validation cohort consisted of 3180 patients. The mean age was 72 years and the median baseline NIHSS score was 16. In total, 188/3180 (5.9%) patients had an sICH (Table 2).

**Table 2.**
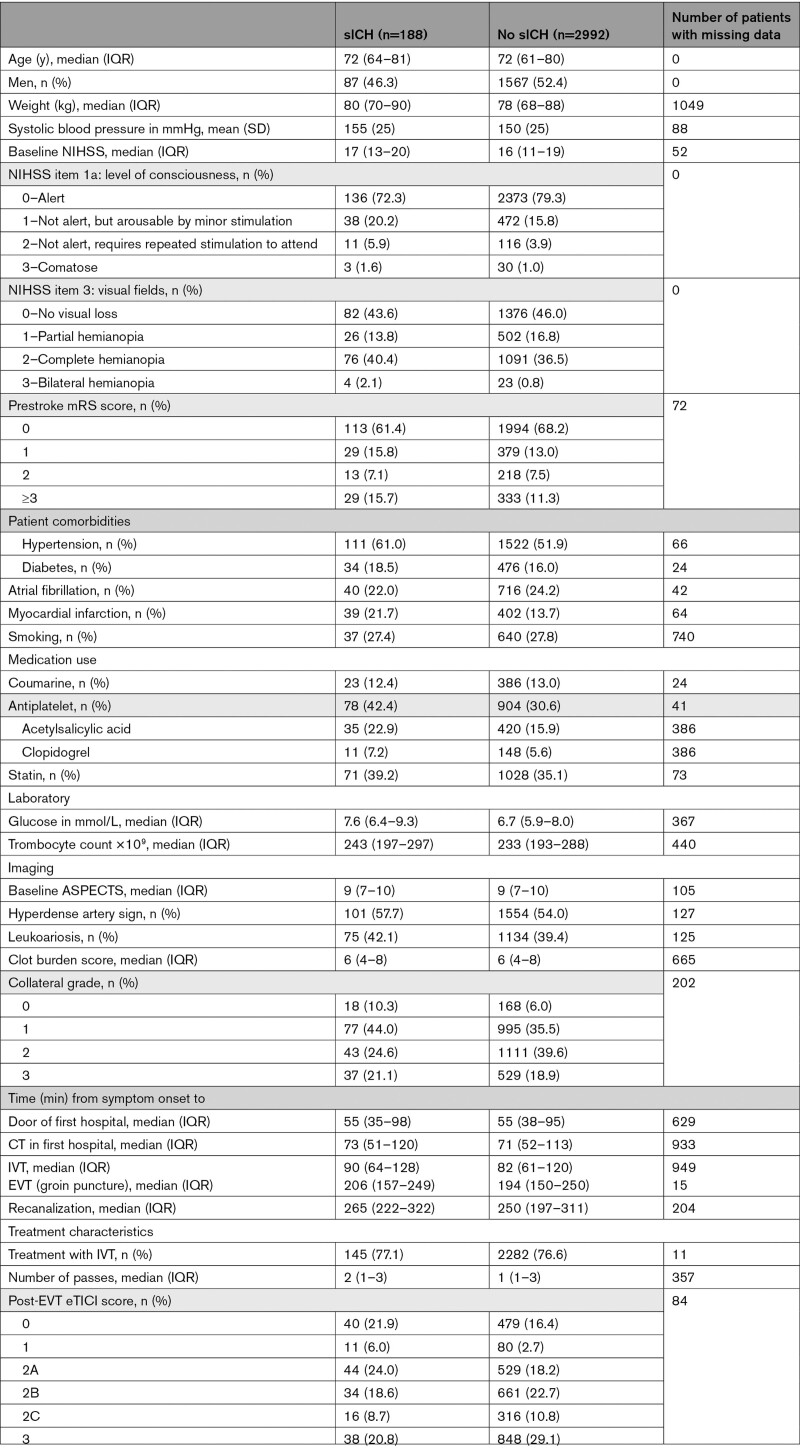
Patient Characteristics in the Validation Cohort According to the Occurrence of Symptomatic Intracranial Hemorrhage

**Figure 2.**
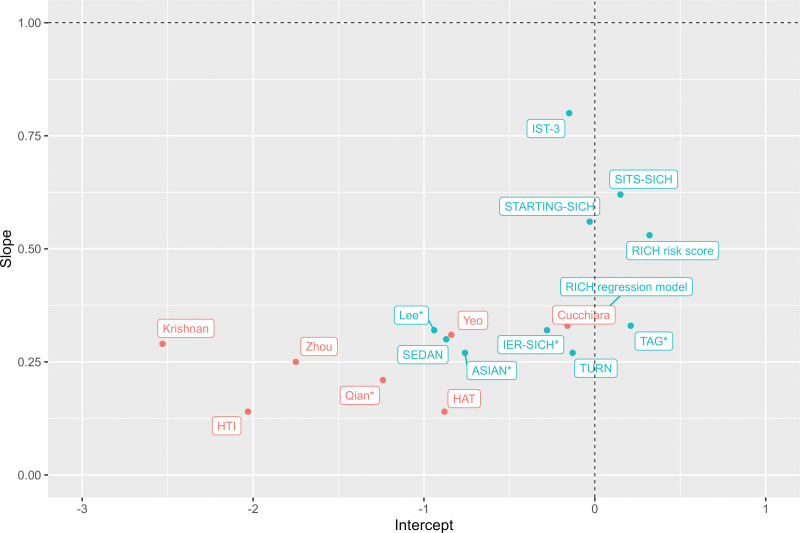
**Calibration intercept and slope of the models in the MR CLEAN Registry (Multicenter Randomized Clinical Trial of Endovascular Treatment for Acute Ischemic Stroke in the Netherlands).** The models in blue are developed to predict symptomatic intracranial hemorrhage (sICH), and the models in red are developed to predict intracranial hemorrhage. The vertical dotted line indicates the ideal calibration intercept and the horizontal dotted line indicates the perfect calibration slope. A calibration intercept >0 indicates systematic underestimation of sICH risk, and an intercept<0 indicates systematic overestimation of sICH risk. A calibration slope <1 reflects that predictions were too extreme: low predictions too low, and high predictions too high. *Indicates models that are developed in a cohort of patients treated with endovascular thrombectomy. For SITS-SICH (Safe Implementation of Treatments in Stroke Symptomatic Intracerebral Hemorrhage), predicted probabilities according to several definitions of sICH are available in the article. In this Figure, calibration was based on predicted probabilities according to sICH per ECASS II (European Cooperative Acute Stroke Study) because this percentage of hemorrhages approximated the percentage in our dataset most and we therefore expected best calibration of the model with these predicted probabilities.

The discriminative ability of the models, expressed as c statistic, ranged from 0.51 (SPAN-100) to 0.61 (SITS-SICH [Safe Implementation of Treatments in Stroke Symptomatic Intracerebral Hemorrhage] and STARTING-SICH [STARTING Symptomatic Intracerebral Hemorrhage]) (Table 3). Model calibration, reported as calibration intercept and slope, varied substantially between studies (Figure 2, Table S4). The values at the extremes of the range for calibration intercept and slope were mainly models developed to predict ICH. Models with the best calibration characteristics were all models developed in a population of patients treated with IVT alone: IST-3 (intercept −0.15 [95% CI, −0.01 to −0.31]; slope 0.80 [95% CI, 0.50–1.09]), SITS-SICH (intercept 0.15 [95% CI, −0.01 to 0.30]; slope 0.62 [95% CI, 0.38–0.87]), and STARTING-SICH (intercept −0.03 [95% CI, −0.19 to 0.12]; slope 0.56 [95% CI, 0.35–0.76]) (Table S5, Figure S1).

**Table 3.**
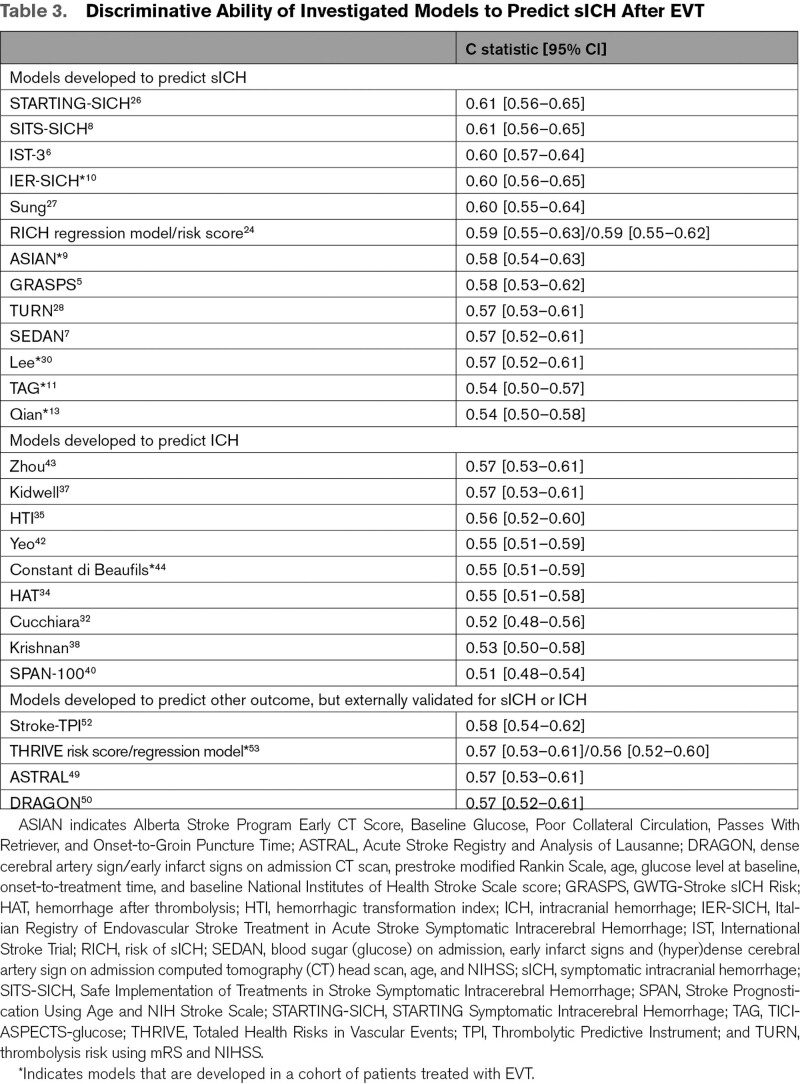
Discriminative Ability of Investigated Models to Predict sICH After EVT

## Discussion

We conducted a systematic search to provide an overview of published models to predict sICH or ICH after reperfusion therapy and externally validated their ability to predict sICH in patients treated with EVT in daily clinical practice. Investigated models to predict sICH or ICH discriminated poorly between patients with a low and high risk of sICH after EVT.

The IST-3, SITS-SICH, and STARTING-SICH showed overall the best predictive performance in terms of discrimination and calibration in stroke patients treated with EVT in daily clinical practice. The models had reasonable calibration characteristics, but the discriminative performance was poor. Even if a model would have perfect calibration characteristics (ie, predicted risk of the outcome for patients is equal to the observed risk), it is useless if it does not discriminate between patients with a low and high risk of the outcome.^12^

The poor discriminative performance of all models can be explained by several reasons. We included models that were developed to predict sICH or ICH according to different definitions, and this may affect strength and nature of the predictors. Another explanation is that most models were developed in patients treated with IVT alone. Although the incidence of sICH in patients treated with IVT alone is similar to patients treated with EVT,^18^ predictors of sICH or the predictive value of predictors may differ. For example, endovascular-procedure–related factors, such as number of passes or reperfusion at the end of the procedure, are important predictors of sICH.^19,20^ Moreover, generalizability of the models might be limited due to different selection criteria for treatment with IVT and/or EVT. Also, the use of antihypertensive medication or antithrombotics could influence the risk of sICH.

The predictive performance of models developed in patients treated with EVT in terms of both discrimination (n=7) and calibration (n=5) was poor. This could be explained because the population in which the models were developed was different from our external validation cohort. For example, TAG was developed in patients treated with EVT within 24 hours and ASIAN was developed in Chinese patients, who are at increased risk of sICH.^9^ Predictors or the prognostic value of predictors in the Asian population may differ from our population. The poor predictive performance of all models in our study emphasizes the importance of external validation.

External validation studies provide the best insight into the performance of a model, indicating how useful it might be in other participants, centers, regions, or settings.^21^ Therefore, it is efficient to evaluate the performance and usefulness of published models before a new model is developed. Because the predictive performance of all models we evaluated was poor, further research should focus on identifying predictors of sICH in patients treated with EVT, including interaction of predictors, before a new model should be developed and externally validated.^12^

Our study has several limitations. First, we evaluated the external validity of models to predict sICH in patients treated with EVT within 6.5 hours of symptom onset. Therefore, we cannot draw any conclusions about the usefulness of these models to predict sICH in patients treated after 6.5 hours of symptom onset or treated with IVT alone. Secondly, some variables were not available in our dataset. Therefore, we were not able to evaluate the external validity of all models: for example, models that included magnetic resonance imaging parameters. For some models, we imputed a missing variable with a value of 0, which might underestimate the performance of a model. For example, GRASPS included Asian ethnicity as predictor, which was not available in our dataset. We assigned all patients a score of 0 (ie, non-Asian ethnicity), because the number of people with Asian ethnicity in the Netherlands is relatively low and we believe this variable could not be imputed based on other variables. However, this might have influenced the predictive performance of GRASPS. Lastly, the predicted outcome in this study was sICH according to the Heidelberg Bleeding Classification. Therefore, the performance of models developed for other outcome measures or other sICH definitions, might be underestimated. However, being able to predict sICH accurately is more relevant than all ICH, because it is more strongly associated with poor functional outcome.

To conclude, the investigated models to predict sICH or ICH discriminate poorly between patients with a low and high risk of sICH after EVT in daily clinical practice. Therefore, these models are not clinically useful for this patient population.

## Article Information

### Acknowledgments

We gratefully appreciate the support of our biomedical information specialist, Wichor M. Bramer, Erasmus MC, who contributed to the literature search. We thank the MR CLEAN Registry investigators listed in Table S5.

### Sources of Funding

The MR CLEAN Registry was partly funded by Stichting Toegepast Wetenschappelijk Instituut voor Neuromodulatie (TWIN), Erasmus MC University Medical Center, Maastricht University Medical Center, and Amsterdam University Medical Center.

### Disclosures

Drs Dippel and van der Lugt report unrestricted grants from Stryker, Penumbra, Medtronic, Cerenovus, Thrombolytic Science, LLC, Dutch Heart Foundation, Brain Foundation Netherlands, The Netherlands Organization for Health Research and Development, and Health Holland Top Sector Life Sciences & Health for research, paid to institution. Dr Majoie received funds from TWIN Foundation (related to this project, paid to institution), CVON/Dutch Heart Foundation, Stryker, European Commission, Health Evaluation Netherlands (unrelated to this project; all paid to institution) and is shareholder of Nicolab. Dr Postma received an institutional grant from Siemens Healthineers. The other authors report no conflicts.

### Supplemental Material

Tables S1–S6

Figure S1

## Supplementary Material
