# Supplementary material for: Symptomatic Intracranial Hemorrhage After Endovascular Stroke Treatment: External Validation of Prediction Models
Source: Stroke. 2023 Jan 24;54(2):476–87. doi: 10.1161/STROKEAHA.122.040065 (PMC9855739; doi:10.1161/STROKEAHA.122.040065)
Supplement: Supplementary file 1 [file str-54-476-s001.pdf]

SUPPLEMENTAL MATERIAL

Symptomatic intracranial hemorrhage after endovascular stroke treatment: External validation of prediction models

Table S1. Search strategy to identify published models to predict symptomatic intracranial hemorrhage.

**Database**	**Search algorithm**
Embase.com	('brain hemorrhage'/exp OR bleeding/de OR 'postoperative hemorrhage'/de OR (hemorrhag* OR haemorrhag* OR microhemorrhag* OR microhaemorrhag* OR bleeding OR lesion*):ab,ti) AND ('thrombectomy'/exp OR 'endovascular surgery'/de OR 'embolectomy'/exp OR 'thrombectomy device'/exp OR 'embolectomy system'/de OR 'blood clot lysis'/exp OR 'fibrinolytic therapy'/exp OR 'fibrinolytic agent'/exp OR 'cerebral revascularization'/de OR 'intraarterial drug administration'/de OR (EVT OR embolect* OR thrombect* OR Soehendra OR Solitaire OR Trevo OR Penumbra OR AngioJet OR APERIO OR ASPIRE OR BONnet OR ((CRC OR pREset OR Revive OR Catch) NEAR/3 (device* OR LITE)) OR ERIC OR FlowTriever OR MindFrame-Capture OR Rotarex OR MERCI OR Phenox-Clot OR ((stent*) NEAR/3 (retriever*)) OR ((thrombus* OR thrombi* OR embol*) NEAR/3 (aspirat* OR excision* OR remov*)) OR ((thrombolys* OR therap* OR treatment* OR procedure*) NEAR/6 (intra-arterial*)) OR endovascular* OR endo-vascular* OR intra-arter* OR intraarter* OR (clot NEAR/3 (lysis OR removal)) OR thromboly* OR postthromboly* OR fibrinoly* OR (plasminogen* NEAR/3 activat*) OR revascular*):ab,ti) AND ('prediction and forecasting'/exp OR 'prognostic assessment'/exp OR 'prognosis'/de OR (predict* OR forecast* OR prognos*):ab,ti) AND ('brain infarction'/exp OR 'cerebrovascular accident'/exp OR (CVA OR stroke* OR ((cerebr* OR brain* OR cerebellum* OR migrain* OR cortical* OR hemispher*) NEAR/3 (infarct*)) OR ((cerebr* OR brain* OR cerebellum*) NEAR/3 (accident* OR lesion* OR vasculopath* OR insult* OR attack* OR disturbance* OR apoplexy* OR apoplec* OR insuffic* OR arrest* OR failure* OR injur*))):ab,ti) NOT ([animals]/lim NOT [humans]/lim) NOT ([Conference Abstract]/lim)
Medline Ovlid	(exp Intracranial Hemorrhages/ OR Hemorrhage/ OR Postoperative Hemorrhage/ OR (hemorrhag* OR haemorrhag* OR microhemorrhag* OR microhaemorrhag* OR bleeding OR lesion*).ab,ti.) AND (exp Thrombectomy/ OR Endovascular Procedures/ OR exp Embolectomy/ OR blood clot lysis/ OR exp Thrombolytic Therapy/ OR exp Fibrinolytic Agents/ OR Cerebral Revascularization/ OR intraarterial drug administration/ OR (EVT OR embolect* OR thrombect* OR Soehendra OR Solitaire OR Trevo OR Penumbra OR AngioJet OR APERIO OR ASPIRE OR BONnet OR ((CRC OR pREset OR Revive OR Catch) ADJ3 (device* OR LITE)) OR ERIC OR FlowTriever OR MindFrame-Capture OR Rotarex OR MERCI OR Phenox-Clot OR ((stent*) ADJ3 (retriever*)) OR ((thrombus* OR thrombi* OR embol*) ADJ3 (aspirat* OR excision* OR remov*)) OR ((thrombolys* OR therap* OR treatment* OR procedure*) ADJ6 (intra-arterial*)) OR

	endovascular* OR endo-vascular* OR intra-arter* OR intraarter* OR (clot ADJ3 (lysis OR removal)) OR thromboly* OR postthromboly* OR fibrinoly* OR (plasminogen* ADJ3 activat*) OR revascular*).ab,ti.) AND (exp Forecasting/ OR Prognosis/ OR (predict* OR forecast* OR prognos*).ab,ti.) AND (exp Brain Infarction/ OR exp Stroke/ OR (CVA OR stroke* OR ((cerebr* OR brain* OR cerebellum* OR migrain* OR cortical* OR hemispher*) ADJ3 (infarct*)) OR ((cerebr* OR brain* OR cerebellum*) ADJ3 (accident* OR lesion* OR vasculopath* OR insult* OR attack* OR disturbance* OR apoplexy* OR apoplec* OR insuffic* OR arrest* OR failure* OR injur*))).ab,ti.) NOT (exp animals/ NOT humans/) NOT (news OR congres* OR abstract* OR book* OR chapter* OR dissertation abstract*).pt.
Cochrane central	((hemorrhag* OR haemorrhag* OR microhemorrhag* OR microhaemorrhag* OR bleeding OR lesion*):ab,ti) AND ((EVT OR embolect* OR thrombect* OR Soehendra OR Solitaire OR Trevo OR Penumbra OR AngioJet OR APERIO OR ASPIRE OR BONnet OR ((CRC OR pREset OR Revive OR Catch) NEAR/3 (device* OR LITE)) OR ERIC OR FlowTriever OR MindFrame next Capture OR Rotarex OR MERCI OR Phenox next Clot OR ((stent*) NEAR/3 (retriever*)) OR ((thrombus* OR thrombi* OR embol*) NEAR/3 (aspirat* OR excision* OR remov*)) OR ((thrombolys* OR therap* OR treatment* OR procedure*) NEAR/6 (intra next arterial*)) OR endovascular* OR endo next vascular* OR intra next arter* OR intraarter* OR (clot NEAR/3 (lysis OR removal)) OR thromboly* OR postthromboly* OR fibrinoly* OR (plasminogen* NEAR/3 activat*) OR revascular*):ab,ti) AND ((predict* OR forecast* OR prognos*):ab,ti) AND ((CVA OR stroke* OR ((cerebr* OR brain* OR cerebellum* OR migrain* OR cortical* OR hemispher*) NEAR/3 (infarct*)) OR ((cerebr* OR brain* OR cerebellum*) NEAR/3 (accident* OR lesion* OR vasculopath* OR insult* OR attack* OR disturbance* OR apoplexy* OR apoplec* OR insuffic* OR arrest* OR failure* OR injur*))):ab,ti)
Web of science	TS=(((hemorrhag* OR haemorrhag* OR microhemorrhag* OR microhaemorrhag* OR bleeding OR lesion*)) AND ((EVT OR embolect* OR thrombect* OR Soehendra OR Solitaire OR Trevo OR Penumbra OR AngioJet OR APERIO OR ASPIRE OR BONnet OR ((CRC OR pREset OR Revive OR Catch) NEAR/2 (device* OR LITE)) OR ERIC OR FlowTriever OR MindFrame-Capture OR Rotarex OR MERCI OR Phenox-Clot OR ((stent*) NEAR/2 (retriever*)) OR ((thrombus* OR thrombi* OR embol*) NEAR/2 (aspirat* OR excision* OR remov*)) OR ((thrombolys* OR therap* OR treatment* OR procedure*) NEAR/5 (intra-arterial*)) OR endovascular* OR endo-vascular* OR intra-arter* OR intraarter* OR (clot NEAR/2 (lysis OR removal)) OR thromboly* OR postthromboly* OR fibrinoly* OR (plasminogen* NEAR/2 activat*) OR revascular*)) AND ((predict* OR forecast* OR prognos*)) AND ((CVA OR stroke* OR ((cerebr* OR brain* OR

	cerebellum* OR migrain* OR cortical* OR hemispher*) NEAR/2 (infarct*)) OR ((cerebr* OR brain* OR cerebellum*) NEAR/2 (accident* OR lesion* OR vasculopath* OR insult* OR attack* OR disturbance* OR apoplexy* OR apoplec* OR insuffic* OR arrest* OR failure* OR injur*))))) AND DT=(article) AND LA=(english)

Table S2. STROBE Statement—Checklist of items that should be included in reports of ***cohort studies***.

	Item No	Recommendation	Addressed on page number
**Title and abstract**	1	(*a*) Indicate the study's design with a commonly used term in the title or the abstract	1
		(*b*) Provide in the abstract an informative and balanced summary of what was done and what was found	3
**Introduction**			
Background/rationale	2	Explain the scientific background and rationale for the investigation being reported	4
Objectives	3	State specific objectives, including any prespecified hypotheses	4
**Methods**			
Study design	4	Present key elements of study design early in the paper	5
Setting	5	Describe the setting, locations, and relevant dates, including periods of recruitment, exposure, follow-up, and data collection	5
Participants	6	(*a*) Give the eligibility criteria, and the sources and methods of selection of participants. Describe methods of follow-up	5
		(*b*) For matched studies, give matching criteria and number of exposed and unexposed	
Variables	7	Clearly define all outcomes, exposures, predictors, potential confounders, and effect modifiers. Give diagnostic criteria, if applicable	6
Data sources/ measurement	8*	For each variable of interest, give sources of data and details of methods of assessment (measurement). Describe comparability of assessment methods if there is more than one group	5-6, Suppleme ntal Table IV
Bias	9	Describe any efforts to address potential sources of bias	6
Study size	10	Explain how the study size was arrived at	4
Quantitative variables	11	Explain how quantitative variables were handled in the analyses. If applicable, describe which groupings were chosen and why	6

Statistical methods	12	(*a*) Describe all statistical methods, including those used to control for confounding	6-7
		(*b*) Describe any methods used to examine subgroups and interactions	NA
		(*c*) Explain how missing data were addressed	7
		(*d*) If applicable, explain how loss to follow-up was addressed	NA
		(*e*) Describe any sensitivity analyses	NA
**Results**			
Participants	13*	(a) Report numbers of individuals at each stage of study—eg numbers potentially eligible, examined for eligibility, confirmed eligible, included in the study, completing follow-up, and analysed	8
		(b) Give reasons for non-participation at each stage	NA
		(c) Consider use of a flow diagram	NA
Descriptive data	14*	(a) Give characteristics of study participants (eg demographic, clinical, social) and information on exposures and potential confounders	8, Table 2
		(b) Indicate number of participants with missing data for each variable of interest	Table 1 and 2
		(c) Summarise follow-up time (eg, average and total amount)	NA
Outcome data	15*	Report numbers of outcome events or summary measures over time	8
Main results	16	(*a*) Give unadjusted estimates and, if applicable, confounder-adjusted estimates and their precision (eg, 95% confidence interval). Make clear which confounders were adjusted for and why they were included	8
		(*b*) Report category boundaries when continuous variables were categorized	8
		(*c*) If relevant, consider translating estimates of relative risk into absolute risk for a meaningful time period	NA
Other analyses	17	Report other analyses done—eg analyses of subgroups and interactions, and sensitivity analyses	NA
**Discussion**			
Key results	18	Summarise key results with reference to study objectives	8-10

Limitations	19	Discuss limitations of the study, taking into account sources of potential bias or imprecision. Discuss both direction and magnitude of any potential bias	10
Interpretation	20	Give a cautious overall interpretation of results considering objectives, limitations, multiplicity of analyses, results from similar studies, and other relevant evidence	8-10
Generalisability	21	Discuss the generalisability (external validity) of the study results	8-10
**Other information**			
Funding	22	Give the source of funding and the role of the funders for the present study and, if applicable, for the original study on which the present article is based	11

*Give information separately for exposed and unexposed groups.

Note: An Explanation and Elaboration article discusses each checklist item and gives methodological background and published examples of transparent reporting. The STROBE checklist is best used in conjunction with this article (freely available on the Web sites of PLoS Medicine at http://www.plosmedicine.org/, Annals of Internal Medicine at http://www.annals.org/, and Epidemiology at http://www.epidem.com/). Information on the STROBE Initiative is available at http://www.strobe-statement.org.

Table S3. Development characteristics of published models to predict symptomatic intracranial hemorrhage.

Model name	Predicted outcome	sICH definition	Treatment in derivation cohort	Number of patients with the outcome/number of total patients in derivation cohort	Regression model or risk score	Number of predictors in model
**ASIAN**[9]	sICH	According to the HBC	Patients treated with EVT in the anterior circulation in China.	87/629 (13.8%)	Risk score	5
**Chung**[22]	sICH	According to ECASS II	Patients treated with IVT.	25/331 (7.6%)	Machine learning	5
**GRASPS**[5]	sICH	According to NINDS	Patients treated with IVT (0.9mg/kg) within 3 hours of symptom onset.	496/10242 (4.8%) in total cohort.<br>In derivation cohort n=7171 (70% of total cohort, but number of patients with the outcome is unknown).	Risk score	6
**Guo**[23]	sICH	According to ECASS II	Patients treated with IVT alone.	66/1200 (5.5%)	Nomogram	4
**IER-SICH**[10]	sICH	According to the HBC	Development: Bridging of EVT with IVT within 6	110/988 (11.1%)	Regression model	5

			hours of symptom onset.<br>Validation cohort: direct EVT within 6 hours of symptom onset.			
**IST-3**[6]	sICH	According to ECASS III	Patients were treated with IVT (0.9mg/kg) within 6 hours of symptom onset (intervention group of IST-3).	104/1515 (6.8%)	Regression model	9
**Lee**[30]	sICH	According to NINDS	Patients within 12 hours of stroke onset with an ischemic lesion on diffusion-weighted MRI. Patients could be treated with IVT or EVT.	53/958 (5.5%)	Regression model	6
**Peng**[31]	sICH	According to the HBC	Patients treated with EVT within 6 hours of symptom onset.	37/334 (11.1%)	Nomogram	3
**Qian**[13]	sICH	According to the HBC	Patients treated with EVT.	21/127 (16.5%)	Nomogram	7

**RICH**[24]	sICH	According to ECASS III	Patients were treated with IVT within 4.5 hours of symptom onset.	53/1336 (4.0%)	Regression model and risk score	5
**SEDAN**[7]	sICH	According to ECASS II	Patients were treated with IVT (0.9mg/kg) within 4.5 hours of symptom onset.	68/974 (7.0%)	Risk score	5
**SICH**[25]	sICH	According to ECASS II	Patients were treated with IVT.	95/1172 (8.1%)	Risk score	6
**SITS-SICH**[8]	sICH	According to SITS-MOST	Patients were treated with IVT (SITS-ISTR) within as well as outside license criteria.	$N_{derivation}$=15814<br>$N_{validation}$=15813<br>N_{total}=31627<br>$sICH_{SITS\text{-}MOST}$ (1.8%)<br>$sICH_{ECASS\ II}$ (5.1%)<br>$sICH_{NINDS}$ (7.4%)	Risk score	10
**STARTING-SICH**[26]	sICH	According to ECASS II	Patients were treated with IVT (SITS-ISTR) within as well as outside license criteria.	$sICH_{ECASS\ II}$ 440/12030 (3.7%)<br>$sICH_{SITS\text{-}MOST}$ 207/12030 (1.7%)	Nomogram	10

**Sung[27] – Extension of SITS-SICH**	sICH	According to NINDS, ECASS II, SITS-MOST	Patients treated with IVT (0.7-0.9mg/kg) within 3 hours of symptom onset.	$sICH_{NINDS}$ (7.3%)<br>$sICH_{ECASS\ II}$ (5.3%)<br>$sICH_{SITS\text{-}MOST}$ (3.5%)	Risk score: SITS-SICH with addition of OCSP classification.	11
**TAG[11]**	sICH	According to ECASS III	Patients treated with EVT within 24 hours of symptom onset.	19/578 (3.3%)	Risk score	3
**TURN[28]**	sICH	According to NINDS	Patients treated with IVT (0.9mg/kg) within 3 hours of symptom onset.	12/210 (5.7%)	Regression model	2
**Wang[29]**	sICH	According to ECASS II	Patients treated with IVT.	102/2237 (4.6%)	Machine learning	5
**Constant Dit Beaufils[44]**	ICH	NA	Patients treated with EVT for an anterior circulation large vessel occlusion.	653/1526 (42.8%)	Regression model	7
**Cucchiara[32]**	ICH	NA	Patients treated with IVT within 3 hours of symptom onset.	158/1205 (12.1%)<br>Predicted probabilities for sICH used for calibration (72/1205; 6.0%)	Risk score	4

**El Nawar**[33]	HT	According to ECASS II	Patients treated with IVT.	52/301 (17.3%)	Regression model	6
**Feng**[45]	ICH	NA	Patients treated with EVT.	34/90 (37.8%)	Regression model	3
**HAT**[34]	ICH	NA	Score based on ranking of cumulative ORs of the predictors from a PubMED literature search.<br><br>Predicted probabilities for calibration based on the combined validation cohorts (NINDS+local cohort of patients treated with EVT.		Risk score	3
**HTI**[35]	ICH	NA	Patients with an ischemic stroke in the middle cerebral artery territory admitted within 12 hours of symptom onset.	126/535 (23.6%)	Risk score	4
**Genot-PA**[36]	ICH	NA	Patients treated with IVT within 4.5 hours of symptom onset.	189/885 (22.1%)	Regression model	6
**Kidwell**[37]	ICH	NA	Patients treated with intra-arterial thrombolysis within 6 hours of symptom onset	35/89 (39%)	Regression model	4

			for anterior circulation occlusions and within 24 hours of symptom onset for posterior circulation.			
**Krishnan**[38]	ICH	NA	Patients with an ischemic stroke in the anterior circulation presenting within 4.5 hours of symptom onset.	106/273 (39%)	Regression model	3
**Liu**[46]	HT	NA	Patients within 24 hours of stroke onset. Patients could be treated with IVT or EVT.	179/1207 (14.8%)	Regression model	14
**Nael**[47]	PH	According to ECASS II	Randomized controlled trial evaluating EVT vs control within 8 hours in patients with an ischemic stroke in the anterior circulation (MR RESCUE).	20/83 (24%)	Regression model	2

**Puig[39]**	HT	According to ECASS II	Patients treated with IVT.	37/156 (23.7%)	Regression model	3
**SPAN-100[40]**	ICH	NA	Randomized controlled trial evaluating IVT vs control within 3 hours (NINDS)	68/624 (10.9%)	Risk score	2
**Wu[41]**	HT	Any ICH according to ECASS II	Patients treated with IVT.	16/131 (12.2%)	Nomogram	10
**Yeo[42]**	ICH	NA	Patients treated with IVT.	Derivation ? n=376<br>Validation 175/922 (18.9%)	Nomogram	4
**Yuan[48]**	HT	NA	Patients treated with IVT and/or EVT within 24 hours of symptom onset.	17/76 (22.4%)	Regression model	2
**Zhou[43]**	ICH	NA	Patients treated with IVT within 24 hours of symptom onset.	33/233 (14.2%)	Nomogram	3
**ASTRAL[49]**	Unfavorable outcome	Modified Rankin Score >2 at 3 months.<br>In external validation for sICH: sICH	Derivation: Patients admitted with an ischemic stroke within 24	External validation cohort: 12/210 (5.71%)	Risk score	6

		according to NINDS criteria.	hours of symptom onset.<br><br>External validation: Patients treated with IVT.			
**DRAGON**[50]	Functional outcome (modified Rankin Scale)	In external validation for sICH: sICH according to NINDS criteria.	Derivation: Patients treated with IVT (0.9mg/kg).<br><br>External validation: Patients treated with IVT.	External validation cohort: 12/210 (5.71%)	Risk score	7
**iScore**[51]	Mortality	In external validation: any ICH.[51]	Derivation: Patients seen in the emergency department or admitted to hospital with an ischemic stroke.<br><br>External validation: Randomized controlled trial evaluating IVT vs control within 3 hours (NINDS)	External validation cohort: 68/624 (10.9%)	Risk score	12

**Stroke-TPI**[52]	Functional outcome (modified Rankin Scale)	In external validation for sICH: sICH according to NINDS criteria.	Derivation: Randomized controlled trials evaluating IVT vs control (NINDS, ATLANTIS A and B, ECASS II).<br><br>External validation: Patients treated with IVT.	External validation cohort: 12/210 (5.71%)	Regression model	3
**THRIVE** risk score	Functional outcome and mortality	In external validation: sICH according to NIINDS, ECASS and SITS-MOST.	Derivation: Patients treated with EVT.<br><br>External validation: Patients treated with IVT.	External validation cohort:<br><br>$sICH_{NINDS}$ 468/5970 (7.3%)<br><br>$sICH_{ECASS}$ 296/9146 (4.6%)<br><br>$sICH_{SITS\text{-}MOST}$ 107/6337 (1.7%)<br><br>No predicted probabilities.	Risk score	5
**THRIVE** regression model[53]	Good outcome (mRS 0-2 at 90 days)	In external validation: sICH according to NINDS	Derivation: Patients treated with and without	External validation cohort:<br><br>61/1128 (5.4%)	Regression model	5

			IVT (VISTA and SITS-MOST).<br><br>External validation: Patients treated with IVT.			

ECASS II, indicates European Cooperative Acute Stroke Study II, sICH defined as neurological deterioration of NIHSS ≥ 4 and any hemorrhage on CT; ECASS III, European Cooperative Acute Stroke Study III, sICH defined as any hemorrhage with neurological deterioration, as indicated by an NIHSS score that was higher by ≥ 4 points than the value at baseline or the lowest value at baseline or the lowest value in the first 7 days, or any hemorrhage leading to death. In addition, the hemorrhage must have been identified as the predominant cause of the neurological deterioration; EVT, endovascular treatment; HBC, Heidelberg Bleeding Classification, sICH defined as any intracranial hemorrhage followed by a neurological deterioration that can be attributed to that hemorrhage, defined as an increase of ≥ 4 points on the NIHSS or ≥ 2 points on a specific NIHSS item; HT, hemorrhagic transformation; ICH, intracranial hemorrhage; NA, not applicable; NINDS, National Institute of Neurological Disorders and Stroke, sICH defined as any hemorrhage associated with neurological deterioration, not further defined; OCSP, Oxfordshire Community Stroke Project; PH, parenchymal hemorrhage; sICH, symptomatic ICH; SITS-MOST, Safe Implementation of Thrombolysis in Stroke-Monitoring Study, sICH defined as a local or remote Type 2 parenchymal hemorrhage on imaging 22 to 36 hours after treatment or earlier if the imaging scan was performed due to clinical deterioration combined with a neurological deterioration of ≥ 4 NIHSS points from baseline or from the lowest NIHSS score between baseline and 24 hours, or leading to death. A grading of Type 2 parenchymal hemorrhage for intracranial hemorrhage indicates a coagulum exceeding 30% of the infarct with substantial space occupation.

Table S4. Overview of models included in external validation with adapted predictors because variables were not available in the MR CLEAN Registry database.

Model	Variable that was not available in our dataset	Adaptation
GRASPS[5]	Ethnicity (non-Asian vs. Asian)	This was imputed as 0 (non-Asian) for all patients.
HTI[35]	Atrial fibrillation on electrocardiogram	This was replaced by history of atrial fibrillation.
IER-SICH[10]	Careggi collateral score, range 0 to 4, with higher scores indicating better collateral flow.	In the MR CLEAN Registry, collaterals were scored by Tan, ranging from 0 to 3, with higher scores indicating a better collateral flow. Patients with a collateral score by Tan of 0, 1, 2, and 3 were assigned a Careggi collateral score of 0, 1, 2.5, and 4, respectively.
DRAGON,[50] SITS-SICH,[8] Lee,[30] and STARTING-SICH[26]	Time from symptom onset to treatment with intravenous thrombolysis.	For patients who had not received intravenous thrombolysis, onset to treatment with intravenous thrombolysis time was calculated as the onset to first CT time plus the median CT to intravenous thrombolysis time.
Constant Dit Beaufils[44]	Coronary artery disease	This was replaced by history of myocardial infarction.

Table S5. Calibration of investigated models to predict symptomatic intracranial hemorrhage.

	Calibration	
	Intercept (95% CI)	Slope (95% CI)
**Models developed to predict sICH**		
**ASIAN***[9]	-0.76 (-0.58 to -0.94)	0.27 (0.12 to 0.43)
**GRASPS**[5]	NA	NA
**IER-SICH***[10]	-0.28 (-0.11 to -0.45)	0.32 (0.19 to 0.45)
**IST-3**[6]	-0.15 (-0.01 to -0.31)	0.80 (0.50 to 1.09)
**Lee***[30]	-0.94 (-0.79 to -1.10)	0.32 (0.10 to 0.53)
**Qian***[13]	-1.24 (-1.08 to -1.42)	0.21 (0.00 to 0.40)
**RICH** regression model/ risk score[24]	0.05 (-0.12 to 0.20)/ 0.32 (0.16 to 0.47)	0.36 (0.20 to 0.51)/ 0.53 (0.30 to 0.77)
**SEDAN**[7]	-0.87 (-0.72 to -1.03)	0.30 (0.10 to 0.51)
**SITS-SICH**[8]	0.15 (-0.01 to 0.30)	0.62 (0.38 to 0.87)
**STARTING-SICH**[26]	-0.03 (-0.19 to 0.12)	0.56 (0.35 to 0.76)
**Sung**[27]	NA	NA
**TAG***[11]	0.21 (-0.06 to -0.38)	0.33 (0.04 to 0.60)
**TURN**[28]	-0.13 (-0.30 to 0.02)	0.27 (0.09 to 0.46)
**Models developed to predict ICH**		

**Cucchiara**[32]	-0.16 (-0.00 to -0.32)	0.33 (0.13 to 0.51)
**HAT**[34]	-0.88 (-0.72 to -1.06)	0.14 (0.00 to 0.27)
**HTI**[35]	-2.03 (-1.85 to -2.22)	0.14 (0.03 to 0.26)
**Kidwell**[37]	NA	NA
**Krishnan**[38]	-2.53 (-2.39 to -2.70)	0.29 (-0.12 to 0.67)
**SPAN-100**[40]	NA	NA
**Yeo**[42]	-0.84 (-0.69 to -1.00)	0.31 (0.06 to 0.55)
**Constant di Beaufils***[44]	NA	NA
**Zhou**[43]	-1.75 (-1.59 to -1.93)	0.25 (0.11 to 0.40)

NA indicates not applicable, because calibration could not be assessed due to missing data in articles.

Figure S1. Calibration plots of IST-3 (A), SITS-SICH (B), and STARTING-SICH (C).

A.

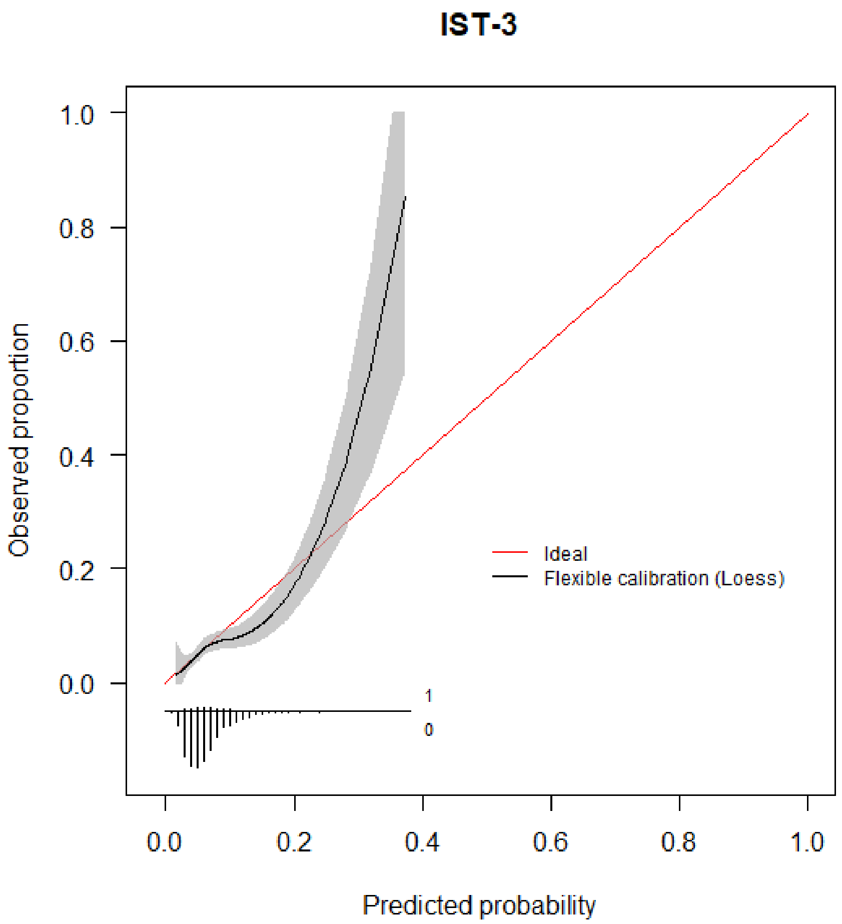

B.

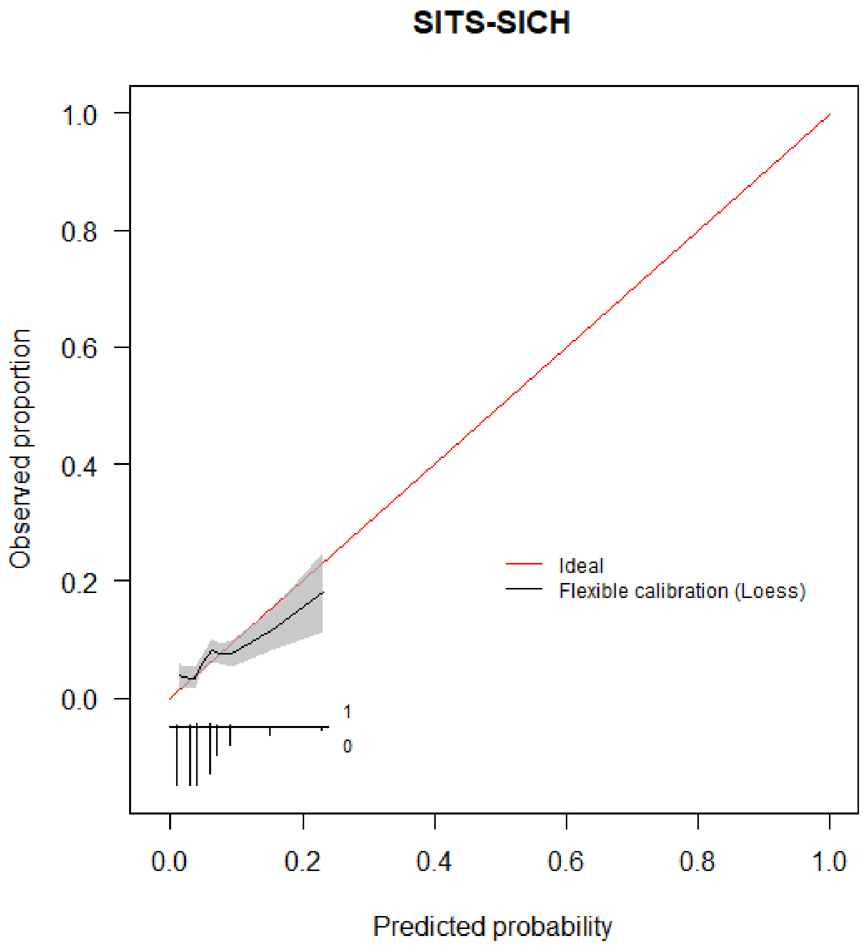
SITS-SICH
1.0
0.8
0.6
0.4
0.2
0.0
Observed proportion
Ideal
Flexible calibration (Loess)
1
0
0.0
0.2
0.4
0.6
0.8
1.0
Predicted probability

C.

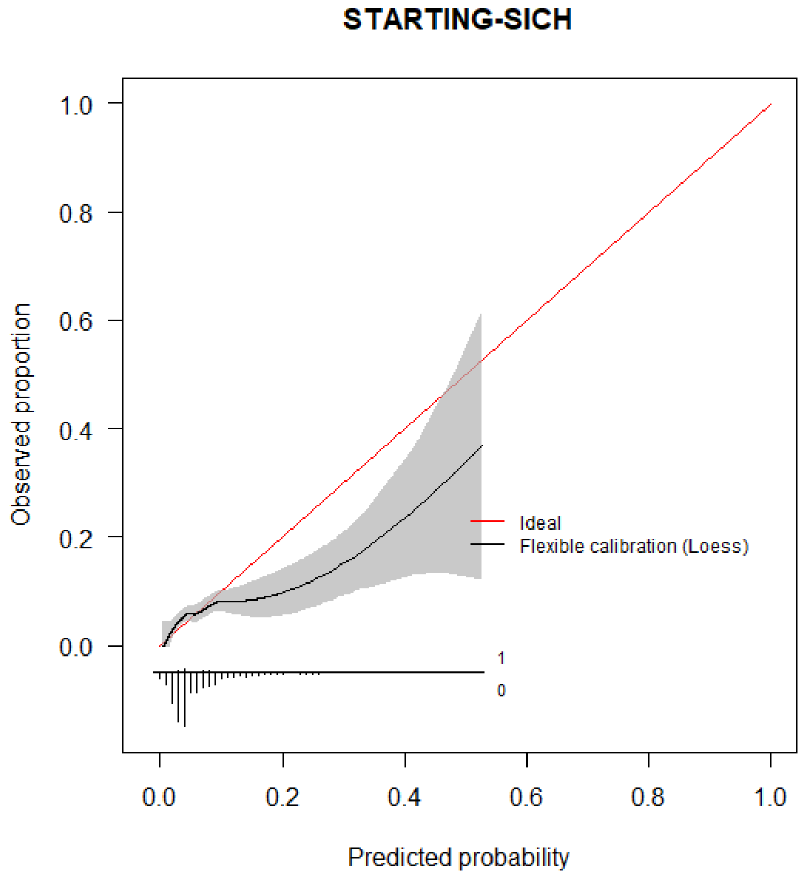
STARTING-SICH
1.0
0.8
0.6
0.4
0.2
0.0
Observed proportion
Ideal
Flexible calibration (Loess)
1
0
0.0
0.2
0.4
0.6
0.8
1.0
Predicted probability

Table S6. MR CLEAN Registry Investigator list.

Executive committee

Diederik W.J. Dippel[1]; Aad van der Lugt[2]; Charles B.L.M. Majoie[3]; Yvo B.W.E.M. Roos[4]; Robert J. van Oostenbrugge[5]; Wim H. van Zwam[6]; Jelis Boiten[14]; Jan Albert Vos[8]

Study coordinators

Ivo G.H. Jansen[3]; Maxim J.H.L. Mulder[1,2] ;Robert- Jan B. Goldhoorn[5,6]; Kars C.J. Compagne[2]; Manon Kappelhof[3]; Josje Brouwer[4]; Sanne J. den Hartog[1,2,40]; Wouter H. Hinsenveld [5,6]

Local principal investigators

Diederik W.J. Dippel[1]; Bob Roozenbeek[1]; Aad van der Lugt[2]; Adriaan C.G.M. van Es[2]; Charles B.L.M. Majoie[3]; Yvo B.W.E.M. Roos[4]; Bart J. Emmer[3]; Jonathan M. Coutinho[4]; Wouter J. Schonewille[7]; Jan Albert Vos[8]; Marieke J.H. Wermer[9]; Marianne A.A. van Walderveen[10]; Julie Staals[5]; Robert J. van Oostenbrugge[5]; Wim H. van Zwam[6]; Jeannette Hofmeijer[11]; Jasper M. Martens[12]; Geert J. Lycklama à Nijeholt[13]; Jelis Boiten[14]; Sebastiaan F. de Bruijn[15]; Lukas C. van Dijk[16]; H. Bart van der Worp[17]; Rob H. Lo[18]; Ewoud J. van Dijk[19]; Hieronymus D. Boogaarts[20]; J. de Vries[22]; Paul L.M. de Kort[21]; Julia van Tuijl[21] ; Jo P. Peluso[26]; Puck Fransen[22]; Jan S.P. van den Berg[22]; Boudewijn A.A.M. van Hasselt[23]; Leo A.M. Aerden[24]; René J. Dallinga[25]; Maarten Uyttenboogaart[28]; Omid Eschgi[29]; Reinoud P.H. Bokkers[29]; Tobien H.C.M.L. Schreuder[30]; Roel J.J. Heijboer[31]; Koos Keizer[32]; Lonneke S.F. Yo[33]; Heleen M. den Hertog[22]; Tomas Bulut[35]; Paul J.A.M. Brouwers[34]

Imaging assessment committee

Charles B.L.M. Majoie[3](chair); Wim H. van Zwam[6]; Aad van der Lugt[2]; Geert J. Lycklama à Nijeholt[13]; Marianne A.A. van Walderveen[10]; Marieke E.S. Sprengers[3]; Sjoerd F.M. Jenniskens[27]; René van den Berg[3]; Albert J. Yoo[38]; Ludo F.M. Beenen[3]; Alida A. Postma[6];

Stefan D. Roosendaal[3]; Bas F.W. van der Kallen[13]; Ido R. van den Wijngaard[13]; Adriaan C.G.M. van Es[2]; Bart J. Emmer[3]; Jasper M. Martens[12]; Lonneke S.F. Yo[33]; Jan Albert Vos[8]; Joost Bot[36]; Pieter-Jan van Doormaal[2]; Anton Meijer[27]; Elyas Ghariq[13]; Reinoud P.H. Bokkers[29]; Marc P. van Proosdij[37]; G. Menno Krietemeijer[33]; Jo P. Peluso[26]; Hieronymus D. Boogaarts[20]; Rob Lo[18]; Dick Gerrits[35]; Wouter Dinkelaar[2]; Auke P.A. Appelman[29]; Bas Hammer[16]; Sjoert Pegge[27]; Anouk van der Hoorn[29]; Saman Vinke[20].

Writing committee

Diederik W.J. Dippel[1](chair); Aad van der Lugt[2]; Charles B.L.M. Majoie[3]; Yvo B.W.E.M. Roos[4] ;Robert J. van Oostenbrugge[5]; Wim H. van Zwam[6]; Geert J. Lycklama à Nijeholt[13]; Jelis Boiten[14]; Jan Albert Vos[8]; Wouter J. Schonewille[7]; Jeannette Hofmeijer[11]; Jasper M. Martens[12]; H. Bart van der Worp[17]; Rob H. Lo[18]

Adverse event committee

Robert J. van Oostenbrugge[5](chair); Jeannette Hofmeijer[11]; H. Zwenneke Flach[23]

Trial methodologist

Hester F. Lingsma[40]

Research nurses / local trial coordinators

Naziha el Ghannouti[1]; Martin Sterrenberg[1]; Wilma Pellikaan[7]; Rita Sprengers[4]; Marjan Elfrink[11]; Michelle Simons[11]; Marjolein Vossers[12]; Joke de Meris[14]; Tamara Vermeulen[14]; Annet Geerlings[19]; Gina van Vemde[22]; Tiny Simons[30]; Gert Messchendorp[28]; Nynke Nicolaij[28]; Hester Bongenaar[32]; Karin Bodde[24]; Sandra Kleijn[34]; Jasmijn Lodico[34]; Hanneke Droste[34]; Maureen Wollaert[5]; Sabrina Verheesen[5]; D. Jeurrissen[5]; Erna Bos[9]; Yvonne

Drabbe[15]; Michelle Sandiman[15]; Nicoline Aaldering[11]; Berber Zweedijk[17]; Jocova Vervoort[21]; Eva Ponjee[22]; Sharon Romviel[19]; Karin Kanselaar[19]; Denn Barning[10].

PhD / Medical students:

Esmee Venema[40]; Vicky Chalos[1,40]; Ralph R. Geuskens[3]; Tim van Straaten[19];Saliha Ergezen[1]; Roger R.M. Harmsma[1]; Daan Muijres[1]; Anouk de Jong[1]; Olvert A. Berkhemer[1,3,6]; Anna M.M. Boers[3,39]; J. Huguet[3]; P.F.C. Groot[3]; Marieke A. Mens[3]; Katinka R. van Kranendonk[3]; Kilian M. Treurniet[3]; Manon L. Tolhuisen[3,39]; Heitor Alves[3]; Annick J. Weterings[3]; Eleonora L.F. Kirkels[3]; Eva J.H.F. Voogd[11]; Lieve M. Schupp[3]; Sabine Collette[28,29]; Adrien E.D. Groot[4]; Natalie E. LeCouffe[4]; Praneeta R. Konduri[39]; Haryadi Prasetya[39]; Nerea Arrarte-Terreros[39]; Lucas A. Ramos[39].

List of affiliations

Department of Neurology[1], Radiology[2], Public Health[40], Erasmus MC University Medical Center;

Department of Radiology[3], Neurology[4], Biomedical Engineering & Physics[39], Amsterdam UMC, University of Amsterdam, Amsterdam;

Department of Neurology[5], Radiology[6], Maastricht University Medical Center and Cardiovascular Research Institute Maastricht (CARIM);

Department of Neurology[7], Radiology[8], Sint Antonius Hospital, Nieuwegein;

Department of Neurology[9], Radiology[10], Leiden University Medical Center;

Department of Neurology[11], Radiology[12], Rijnstate Hospital, Arnhem;

Department of Radiology[13], Neurology[14], Haaglanden MC, the Hague;

Department of Neurology[15], Radiology[16], HAGA Hospital, the Hague;

Department of Neurology[17], Radiology[18], University Medical Center Utrecht;

Department of Neurology[19], Neurosurgery[20], Radiology[27], Radboud University Medical Center, Nijmegen;

Department of Neurology[21], Radiology[26], Elisabeth-TweeSteden ziekenhuis, Tilburg;

Department of Neurology[22], Radiology[23], Isala Klinieken, Zwolle;

Department of Neurology[24], Radiology[25], Reinier de Graaf Gasthuis, Delft;

Department of Neurology[28], Radiology[29], University Medical Center Groningen;

Department of Neurology[30], Radiology[31], Atrium Medical Center, Heerlen;

Department of Neurology[32], Radiology[33], Catharina Hospital, Eindhoven;

Department of Neurology[34], Radiology[35], Medical Spectrum Twente, Enschede;

Department of Radiology[36], Amsterdam UMC, Vrije Universiteit van Amsterdam, Amsterdam;

Department of Radiology[37], Noordwest Ziekenhuisgroep, Alkmaar;

Department of Radiology[38], Texas Stroke Institute, Texas, United States of America.
